# Do the Microshear Test Variables Affect the Bond Strength Values?

**DOI:** 10.1155/2012/618960

**Published:** 2012-11-04

**Authors:** Andrea M. Andrade, Eugenio Garcia, Sandra Kiss Moura, Alessandra Reis, Alessandro Loguercio, Luciana Mendonça Silva, Gustavo H. D. Pimentel, Rosa Helena Miranda Grande

**Affiliations:** ^1^Department of Biomaterials and Oral Biology, School of Dentistry, University of São Paulo, Cidade Universitária, 05508-000 São Paulo, SP, Brazil; ^2^Department of Restorative Dentistry, School of Dentistry, University of North of Parana, Rua Marselha 183, Jardim Piza, 86041-140 Londrina, PR, Brazil; ^3^Department of Restorative Dentistry, School of Dentistry, State University of Ponta Grossa, Avenue Carlos Cavalacanti 4748, Uvaranas, 84030-900 Ponta Grossa, PR, Brazil; ^4^School of Dentistry, Federal University of Amazonas, Avenue Ministro Valdemar Pedrosa 1539, Centro, 69025-050 Manaus, AM, Brazil

## Abstract

Little is known about the effect of specimen preparation and testing protocols on the micro-shear bond strength (**μ**SBS) results. To evaluate whether variations in polyethylene rod use affect (**μ**SBS)). Human dentin disks were randomly distributed into six groups (*n* = 5): polyethylene tube (3 levels) and adhesive system (2 levels). In Group 1, polyethylene tubes filled with polymerized composite) were placed on adhesive covered surfaces. Tubes were removed 24 h after water storage, leaving the rods only. In Group 2, the same procedure was performed; however, tubes were kept in place during testing. In Group 3, composite rods without tubes were placed on adhesive covered dentin. In all groups, adhesives were photoactivated after positioning filled tubes/rods on adhesive covered surfaces. Specimens were tested under shear mode and the data subjected to a two-way ANOVA and Tukey's tests. Groups 1 and 2 resulted in statistically similar mean **μ**SBS (*P* > 0.05); however, a greater number of pretest failures were observed for Group 1. Higher **μ**SBS values were detected for Group 3, irrespective of adhesive system used (*P* < 0.05). Removing the polyethylene tube before composite rod is placed on dentin affects **μ**SBS values.

## 1. Introduction

Bond strength measurement is one of the most common methods for evaluating the adhesive properties of restorative materials. Various mechanical methods, such as tensile, microtensile, flexural, shear, and in-plane shear tests have been used to assess bond to dental substrate [1, 2].

Compared with conventional tensile and shear tests both microtensile and microshear tests allow standard tooth regions to be selected, thus preserving the uniformity of the testing area [3, 4]. The simple test protocol of the microshear test [5, 6] allows for straightforward specimen preparation. It also permits regional mapping of substrate surfaces and depth profiling of the substrate [5, 6]. This means that the *μ*SBS test could have additional advantages over the *μ*TBS test, because it is performed without the need for sectioning procedures, which may induce early microcracking, to obtain specimens [1, 2].

Although sequential sectioning is unnecessary to obtain specimens for microshear testing [6, 7], a polyethylene tube is used as a mold for composite placement. However, similar to data reported for the macro-shear test, this can lead to the introduction of flaws and different stress concentrations under shear loading [8]. Moreover, in the majority of studies, the polyethylene tubes are removed with a scalpel blade before testing [3–5, 7, 9, 10], which may lead to stress at the adhesive interface and result in premature failures. This has led to some authors keeping the polyethylene tubes in position for testing [11]. However, to the extent of the authors' knowledge, no study has so far polyethylene tubes filled with prepolymerized composite for testing resin-dentin bond strength produced by different materials and techniques by means of the microshear approach. 

Contrary to the large number of *μ*TBS studies analyzing the influence of test parameters on the bond strength values [1, 12, 13], few microshear test studies have been conducted with this aim [7, 10, 11, 14]. Therefore, it might be relevant to study variations in the methodology regarding the use of polyethylene tube and its effect on the resin-dentin bond strength of two two-step etch-and-rinse adhesives.

## 2. Materials and Methods

### 2.1. Teeth Selection and Preparation

A total of 30 human molar teeth were collected after obtaining the approval of the Institutional Ethics Review Board (Protocol 193/06). The teeth were cleaned by removing all debris and stored in a refrigerator in 0.5% chloramine solution for one week. Before preparation, the teeth were removed from the chloramine solution and washed under abundant running water to remove remnants of the solution. 

The crowns of the teeth were transversally removed with a diamond saw at slow speed under water irrigation (Labcut 1010, Extec Corp., Enfield, CT, USA). A second cut was made parallel to the first, in order to obtain 1.5 mm thick mid-coronal dentin disks. Next, the coronal dentin surfaces were examined under a stereomicroscope at 20× magnification (HMW-2, Shimadzu, Tokyo, Japan) to ensure that they were free of enamel remnants. The dentin slices were then polished on wet 600-grit silicon carbide paper for 60 s to standardize the smear layer. After this, the specimens were ultrasonically cleaned in distilled water for 5 min before the bonding procedure to remove the remaining silicon carbide dust particles.

### 2.2. Bonding Procedures

Two etch-and-rinse two-step adhesives (Adper Single Bond 3 M ESPE, St. Paul, MN, USA) and XP Bond (Dentsply, Konstanz, B-W, Germany) and a hybrid composite (Filtek Z250, 3 M ESPE) were used in this study. The dentin was etched with 35% phosphoric acid (3 M ESPE), and the adhesives were applied according to manufacturers' instructions (Table 1). All polymerization procedures were carried out with an Optilux 500 device (Kerr Corp, Orange, CA, USA) with a light output of 600 mW/cm^2^. The teeth were then divided into three groups: in each group, Adper Single Bond 2 and XP Bond were applied and testing, as follows.

In Group 1, composite-filled polyethylene tubes with an adhesive area of 0.44 mm^2^ (TYGON Medical Tubing Formulations 54-HL, Saint Gobain Performance Plastics, Akron, OH, USA) were placed on mylar strips. The tubes were carefully packed with composite and light-polymerized. Six tubes filled with light-polymerized composite were placed on the adhesive-treated dentin surfaces. When the tubes were in place, the adhesives were light-polymerized in accordance with the manufacturer's directions. Specimens were stored in distilled water at 37°C for 24 h. After 24 h, the tubes were removed with a sharp blade, thus obtaining a composite rod. The flash of composite resin extending beyond the base of the composite resin rod was also cut off with a sharp blade.

In Group 2, all procedures described in Group 1 were repeated, except that the polyethylene tubes were not removed for testing. In Group 3, the same procedures described for Group 1 were repeated, except that the polyethylene tubes were removed with a sharp blade first, only the polymerized composite rods were placed on the adhesive-covered dentin, and then the adhesives were photoactivated. 

### 2.3. Shear Bond Strength Testing

Before testing, specimens were checked with a light stereomicroscope at 10× magnification to discard specimen with presence of air bubbles or gaps at the interface. Each dentin slice was fixed in a PVC (polyvinyl chloride) tube previously filled with acrylic resin. The PVC tube with the specimen was taken to the testing device *Bisco Shear Bond Tester* (Bisco Inc., Schaumburg, IL, USA). Each tube was subjected to a *μ*SBS test using a semicircular metal attachment, which was placed as close as possible to the composite/dentin interface. The test was run at a crosshead speed of 1.0 mm/min. The force required for failure (Newton) was divided by the surface area (mm^2^) to calculate the shear bond strength in MPa.

### 2.4. SEM Evaluation

After microshear testing, both sides of all fractured specimens were mounted on aluminum stubs, sputter-coated, and observed by scanning electron microscopy (JEOL 5600 LVj, JEOL Ltd., Tokyo, Japan). The bond failure modes were evaluated and classified as one of three types: mixed (adhesive + cohesive failure of the neighboring substrates), cohesive (failure exclusively within dentin or resin composite), and adhesive (failure exclusively at the adhesive interface or within the bonding material). 

### 2.5. Statistical Analysis

Five teeth were used for each experimental condition. The bond strength values of all specimens from the same were averaged for statistical purposes. The pretest failures (PTFs) were either included in the tooth mean, or not. In each case (with and without the inclusion of the PTF values), the data were subjected to a two-way analysis of variance (*α* = 0.05). For all analyses, pairwise comparisons were made using the Tukey's post hoc test (*α* = 0.05). The number of PTF was evaluated with Fisher's exact test (*α* = 0.05).

## 3. Results

**Figure 1:**
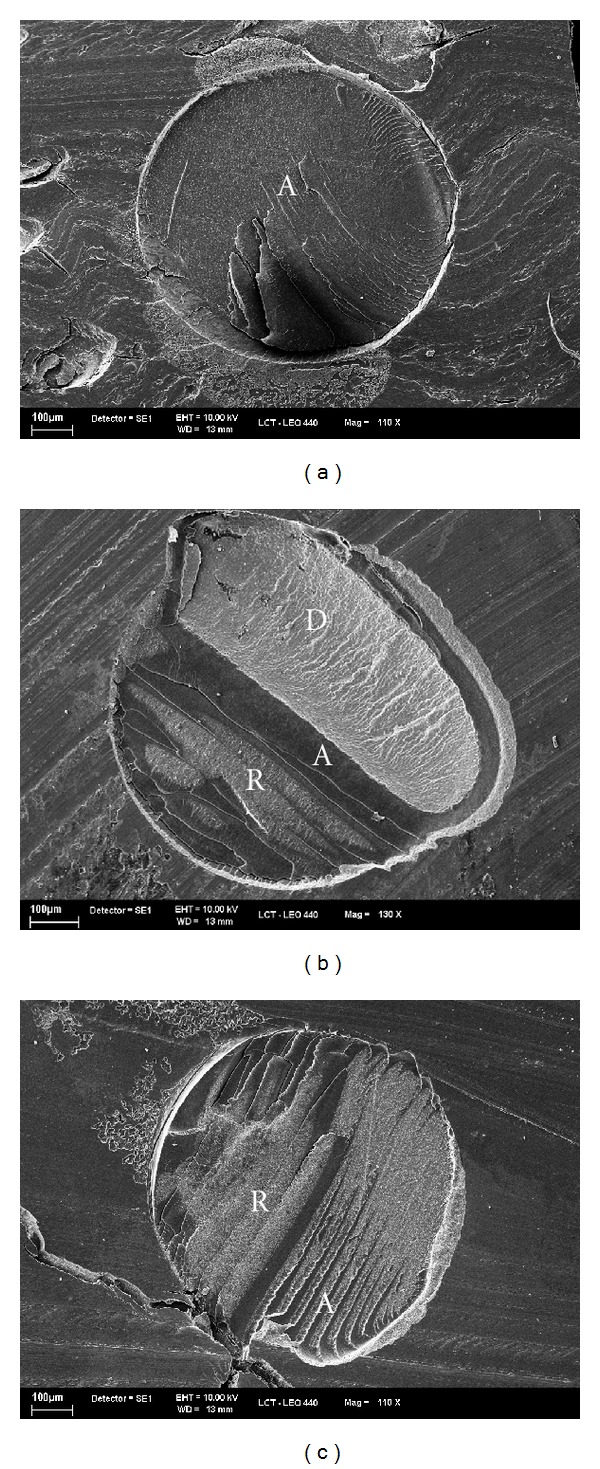
SEM photomicrographs showed adhesive (a) failure and mixed failure (b) and (c). In (b), observe a mixed failure between adhesive (A), dentin (D), and composite resin (R) and in (c) a mixed failure between composite resin (R) and adhesive (A).

No cohesive failures were observed in the present investigation. The majority of the fracture patterns observed were adhesive (72.2%) and mixed (11.7%) (Figure 1). With regard to PTF, significant differences were observed for groups (*P* < 0.05). A higher number of PTF was observed when the polyethylene tube was removed after 24 h (24% and 30.8%, resp., for SB and XP), in comparison with Groups 2 and 3. In Group 3 no PTF was recorded (*P* < 0.05) (Table 2).

The mean *μ*SBS values and the respective standard deviations are shown in Table 3. The results of the two-way ANOVA revealed that the cross-product interaction was statistically significant (*P* < 0.05) for both analyses (with or without PTF). In both analyses, higher bond strength values were observed for Group 3 (*P* < 0.05) irrespective of the adhesive system (Table 3). 

## 4. Discussion

The microshear test is a relatively simple “micro” test that permits efficient screening of adhesive systems, regional and depth profiling of a variety of substrates and conservation of teeth [15]. Some authors argue that the *μ*SBS method presents a significant advantage over microtensile bond strength methods, since the *μ*SBS specimen is not prestressed by specimen sectioning prior to testing [8]. However this advantage is only partially true. Most microshear studies use polyethylene tubes as molds, which are then filled with a resin composite. After water storage for 24 h, the operator uses a scalpel blade to remove these tubes manually, resulting in cylindrical composite specimens [3, 5, 7, 9, 10]. The pressure exerted on the blade by the operator in order to cut and remove the polyethylene tubes may be transferred to the resin cylinder and consequently form cracks along the specimen. Therefore, it is fair to hypothesize that microshear specimens may fail under relatively low loading levels or fail prematurely due to propagation of these cracks [10]. 

Usually, no comments are made about pretest failures in the microshear bond strength test; however it could be considered the factor responsible for the different number of specimens for each group in each study [6, 11, 16].

In the present investigation, it was observed that removal of the Tygon tubes before testing did not seem to affect the bond strength of the adhesive systems tested. This finding was also mentioned in the other study [11], which reported that no difference in terms of bond strength to enamel was found in a pilot study, in which the Tygon tubes were either kept or removed for load application. At a first glance, one could conclude that either keeping or removing the Tygon tubes did not affect the outcomes of any experiment with microshear testing. However, a closer look at the number of PTF indicated that even when the Tygon tubes were carefully removed, some level of stress was induced at the interface, since the tube removal procedure yielded a high number of pretest failures, in addition to showing an effect on the microtensile bond strength test [1, 2].

On the other hand, one cannot say that the Tygon tube should be left in place for testing. Finite element analysis has shown that the nominal bond strength measured for the same material could change with variations in specimen geometries, loading configurations, or material stiffness because of differences in stress distribution at the bonded interface [17]. The stress distribution during shear loading on the composite rods enclosed within a polyethylene tube is not known, since the polyethylene tube is a resilient material capable of absorbing some of the stresses produced during shear loading. It was observed that the use of low modulus composites may influence stress concentration [10, 14]. 

In view of this, the aim of this study was to investigate whether removal of the polyethylene tubes before the composite rod was placed on the dentin surface could affect the results obtained. Higher resin-dentin bond strength values and no PTF were observed, which indicated that either keeping or removing the Tygon affects the range of bond strength values that can be detected by means of microshear testing.

It is worth mentioning that any bond strength testing method should be able to detect differences among materials/techniques in order to allow the screening and ranking of materials/techniques. The ranking and the resin-dentin bond strength values of some adhesives gathered from bond strength studies, published in the last ten years, were shown to be quite similar when microshear and microtensile bond strength testing methods were used [4, 10, 16], Moreover, micro-shear method was shown to have the advantage of producing fewer cohesive failures and presenting lower data dispersion [2]. 

Therefore, bond strength tests capable of detecting high bond strength values are preferable, since the higher the bond strength values measured, the higher is the sensitivity of the method in detecting subtle differences among materials and techniques. Therefore researchers should consider the use of prepolymerized composite rods in future micro-shear tests.

A clear disadvantage of this novel approach is that the bond strength measured does not take into consideration the effects of polymerization shrinkage of the composite resin when light polymerized in contact with the adhesive system, and one cannot rule out the fact that this may have played a role in the higher bond strength values observed for this group [18]. However, this does not seem to be main reason for the higher bond strength values, since previous literature findings have shown that the stress produced by polymerization shrinkage is not high when bonding is performed in a low-constraint situation such as a flat dentin surface [18, 19].

Thus, one can say that the better performance of Group 3 may be due to lack of crack formation caused by either removal of the Tygon tube or an uneven stress distribution when the polyethylene tube is left in place. Crack formation [8] may have been initiated in the resin cylinder during tube removal because this material is more brittle in comparison with the dentin surface and brittle materials are expected to have a higher speed of crack speed propagation. According to the Griffith's theory, a fracture is a very complex process that involves the nucleation and growth of microvoids or cracks and their displacement and propagation.

Different materials were used to evaluate the microshear bond strength values under the experimental conditions of this study. The choice of these materials was based on the fact that they perform well in terms of interfacial strength, so that their performance would not compromise the investigation of this experimental condition of this study [20, 21]. Although some small differences were detected in terms of bond strength values between adhesives, the overall conclusions of the experimental conditions were the same, showing that the highest bond strength values were yielded when the composite rod was placed directly on the dentin surface without any polyethylene tube. 

According to fracture mode evaluation, most of the failures were adhesive or mixed irrespective of the experimental groups. This is an additional advantage of the microshear test. In addition to the fact that no sequential sectioning is required to prepare the specimens, this test greatly reduces the occurrence of cohesive substrate fractures [1, 9]. 

## 5. Conclusions

One may conclude that either keeping or removing the Tygon tube for *μ*SBS testing reduces the range of *μ*SBS values produced by the test, that is, the test sensitivity to detect subtle differences between groups. This novel approach yields a more appropriate screening of materials/technique due to the higher range of *μ*SBS values detected.

## Figures and Tables

**Table 1:** Materials, batch number, composition, and mode of application.

Materials/batch number	Composition	Mode of application
Adper single bond [7 MR]	Bis-GMA, HEMA, dimethacrylates, polyalkenoic acid copolymer, initiators, water, and ethanol	(1) Apply phosphoric acid to dentin for 15 s
		(2) Rinse for 15 s
		(3) Keep dentin wet
		(4) Apply two consecutive coats of adhesive for 15 s with gently agitation
		(5) Gently air for 5 s to evaporate the solvent (10 cm, 45° inclination with 1-bar pressure)
		(6) Light polymerize for 10 s

XP Bond [0710004024]	TCB-resin, PENTA,UDMA, TEGDMA, HEMA, butylated benzenediol, ethyl-4-dimethylaminobenzoate, camphorquinone, nanofiller, t-butanol	(1) Apply phosphoric acid to dentin for 15 s
		(2) Rinse for 15 s
		(3) Apply the adhesive, and leave undisturbed for 20 seconds
		(4) Gently air for 5 s to evaporate the solvent (10 cm, 45° inclination with 1-bar pressure)
		(5) Light polymerize for 20 s

Filtek Z250 [7 WN]	(1) Filler type—zirconia, silica	(1) Light polymerize each increment for 40 s
	(2) Resin—Bis-GMA, UDMA, and Bis-EMA	

Bis-GMA: 2,2-bis[4-(2-hydroxy-3-methacryloyloxypropoxy)]-phenyl propane; HEMA: 2-hydroxyethylmethacrylate; TEGDMA: tryethyleneglycol dimethacrylate; UDMA: urethane dimethacrylate; TCB-resin: carboxylic acid-modified dimethacrylate; PENTA: phosphoric acid-modified acrylate resin; Bis-EMA: bisphenol-A, ethoxylate 0110 dimethacrylate.

**Table 2:** Number of specimens (%) distributed according to the failure pattern for each experimental condition (*).

Adhesive systems	Groups	Pattern failure (%)				
		Adhesive	Mixed	Cohesive		PTF
				Resin	Dentin
Adper	1	14 (56.0)	5 (20.0)	0 (0)	0 (0)	6 (24.0)
Single	2	22 (91.7)	2 (8.3)	0 (0)	0 (0)	0 (0)
Bond	3	15 (50.0)	15 (50.0)	0 (0)	0 (0)	0 (0)

XP Bond	1	18 (69.2)	0 (0)	0 (0)	0 (0)	8 (30.8)
	2	26 (92.9)	0 (0)	0 (0)	0 (0)	2 (7.1)
	3	22 (73.3)	8 (26.7)	0 (0)	0 (0)	0 (0)

**Table 3:** Mean bond values and standard deviation (MPa) with and without PTF and according to each experimental condition (*).

Adhesive	Groups	PTF inclusion	PTF exclusion
Adper single bond	1	14.0 ± 5.7^d^	14.8 ± 3.1^D^
	2	18.1 ± 3.6^c, d^	18.1 ± 3.6^C, D^
	3	37.7 ± 3.7^a^	37.7 ± 3.7^A^

XP bond	1	14.7 ± 4.2^d^	16.2 ± 4.2^C, D^
	2	19.7 ± 1.6^c^	20.5 ± 1.1^C^
	3	26.0 ± 4.4^b^	26.0 ± 4.4^B^

(*) Lowercase letters indicated significant difference between “pretest (PTF) failure inclusion” (*P* < 0.05); capital letters indicated significant difference between “PTF exclusion” (*P* < 0.05).
